# PI(4,5)P2-dependent and -independent roles of PI4P in the control of hormone secretion by pituitary cells

**DOI:** 10.3389/fendo.2023.1118744

**Published:** 2023-01-27

**Authors:** Stanko S. Stojilkovic, Tamas Balla

**Affiliations:** ^1^ Section on Cellular Signaling, Eunice Kennedy Shriver National Institute of Child Health and Human Development, National Institutes of Health, Bethesda, MD, United States; ^2^ Section on Molecular Signal Transduction, Eunice Kennedy Shriver National Institute of Child Health and Human Development, National Institutes of Health, Bethesda, MD, United States

**Keywords:** phosphoinositides, PI4P, PI(4,5)P2, PI(3,4,5)P3, lactotrophs, calcium, hormone secretion

## Abstract

Plasma membrane and organelle membranes are home to seven phosphoinositides, an important class of low-abundance anionic signaling lipids that contribute to cellular functions by recruiting cytoplasmic proteins or interacting with the cytoplasmic domains of membrane proteins. Here, we briefly review the functions of three phosphoinositides, PI4P, PI(4,5)P2, and PI(3,4,5)P3, in cellular signaling and exocytosis, focusing on hormone-producing pituitary cells. PI(4,5)P2, acting as a substrate for phospholipase C, plays a key role in the control of pituitary cell functions, including hormone synthesis and secretion. PI(4,5)P2 also acts as a substrate for class I PI3-kinases, leading to the generation of two intracellular messengers, PI(3,4,5)P3 and PI(3,4)P2, which act through their intracellular effectors, including Akt. PI(4,5)P2 can also influence the release of pituitary hormones acting as an intact lipid to regulate ion channel gating and concomitant calcium signaling, as well as the exocytic pathway. Recent findings also show that PI4P is not only a precursor of PI(4,5)P2, but also a key signaling molecule in many cell types, including pituitary cells, where it controls hormone secretion in a PI(4,5)P2-independent manner.

## Introduction

**Figure 1:**
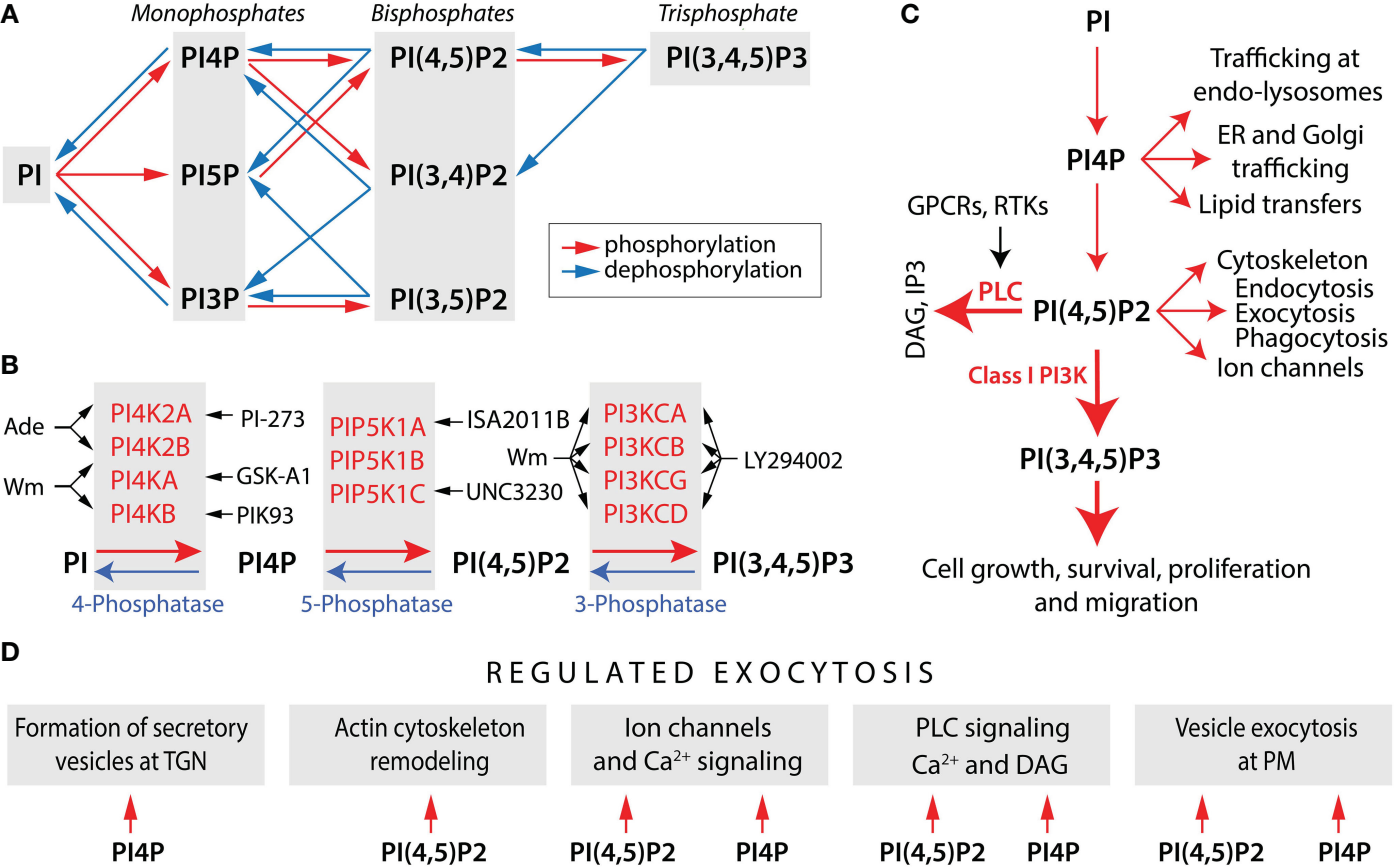
Phosphoinositide metabolism and functions. **(A)** Schematic representation of phosphoinositide metabolism. The concerted actions of PI-kinases (red arrows) and PI-phosphatases (blue arrows) generate three monophosphates, three bisphosphates, and a single trisphosphate. For simplicity, reactions that are likely to predominate *in vivo* are shown. **(B)** PI-kinases and PI-phosphatases involved in the metabolism of PI4P, PI(4,5)P2, and PI (3,4,5) P3 and kinase inhibitors discussed in this review. Black arrows indicate the enzymes inhibited by drugs. Wortmannin (Wm), a fungal metabolite, is a cell-permeable and irreversible inhibitor of phosphatidylinositol 4-kinases PI4KA and PI4KB, both in a micromolar concentration range (1), whereas PI4K2A and PI4K2B are inhibited by adenosine (Ade) (2). PI4KA is specifically inhibited by GSK-A1 and PI4KB by PIK93 (3), whereas PI4K2A is inhibited by PI-273 (4). The activity of PIP5K1A is blocked by ISA2011B (5) and PIP5K1C by UNC3230 (6). PI5P also contribute to the formation of PI (4,5) P2 by PI4K2A/B, which are inhibited by Bay-091 and Bay-297 (7). PI(4,5)P2 is a substrate for the class I PI3-kinases, consisting of PI3KCA, PI3KCB, PI3KCG, and PI3KCD isoforms. The blockers of these enzymes are Wm (in a nanomolar concentration range), LY294002, and a larger number of other inhibitors (8). **(C)** Multiple functions of PI(4,5)P2 and PI4P. Two major actions of PI(4,5)P2 are to serve as a substrate for two signaling pathways: phospholipase C (PLC)-dependent and PI3K-dependent. PLC is activated by Gq/11 protein-coupled receptors (GPCRs) and receptor tyrosine kinases (RTKs), leading to formation of two intracellular messengers: inositol (1,4,5)-trisphosphate (IP3) and diacylglycerol (DAG). PI(3,4,5)P3 exhibits its intracellular messenger functions by binding to numerous PH-domain containing effectors. PI(4,5)P2 also directly regulates ion channel gating, cytoskeleton, and membrane dynamics, including endocytosis, exocytosis, and phagocytosis. PI4P is not only a precursor of PI(4,5)P2, but also acts as an intracellular messenger that controls cellular function independently of PI(4,5)P2, including trafficking at endosomes (endo) and lysosomes, endoplasmic reticulum (ER) and Golgi trafficking, and lipid transfers. **(D)** Common and specific roles of PI4P and PI(4,5)P2 in hormone secretion. The formation of secretory vesicles and carriers at the *trans* Golgi network (TGN) is dependent on PI4P (9). In contrast, PI(4,5)P2 regulates remodeling of actin cytoskeleton (10), which may contribute to secretory vesicle travel. Both PI4P and PI(4,5)P2 can regulate ion channel gating, but differentially (11). In addition to PI(4,5)P2, PI4P is also involved in PLC signaling as a direct substrate. However, PI(4,5)P2 hydrolysis release IP3 and DAG, whereas PI4P hydrolysis release DAG and inactive IP2, an action that could explain the prolonged production of DAG during sustained GPCR activation (12). The last step in vesicle exocytosis was believed to be solely dependent on PI(4,5)P2 (13), but recent data suggested a possible role for PI4P independent of PI(4,5)P2 in the final step of regulated exocytosis (14). Derived from (9, 10, 14–16).

Eukaryotic cells are compartmentalized into organelles and have an extensive endomembrane system that includes the endoplasmic reticulum (ER), nuclear membrane, Golgi apparatus, and lysosomes, in addition to the plasma membrane (PM). The synchronized function of this membrane network is critically dependent on the presence of phosphatidylinositol (PI) as the ultimate precursor of phosphoinositides, also known as PI phosphates (PIPs), a group of signaling and structural lipid molecules involved in numerous cellular processes. This include defining the identity of intracellular organelles, signal transduction, cell survival and proliferation, cytoskeleton organization, membrane trafficking, modulation of gene expression, and hormone/neurotransmitter release. They are a set of seven lipid derivatives that differ in the presence or absence of phosphate groups at the 3-, 4-, and 5-positions of PI. As illustrated in **Figure 1A**, they include the three monophosphates - PI3P, PI4P, and PI5P; the three bisphosphates – PI(4,5)P2, PI(3,5)P2, and PI(3,4)P2; and one trisphosphate – PI(3,4,5)P3. PI-kinases (PIKs) and PI-phosphatases are responsible for the conversion of PIPs between these distinctive phosphorylation states (17).

Here we review the roles of PI4P, PI(4,5)P2, and PI(3,4,5)P3 in pituitary cell signaling and hormone secretion. The pituitary gland is a neuroendocrine organ consisting of six hormone-producing cells (HPCs): corticotrophs that secrete adenocorticotropic hormone, melanotrophs that secrete melanocyte-stimulating hormone and beta-endorphin, gonadotrophs that secrete luteinizing hormone and follicle-stimulating hormone, thyrotrophs that secrete thyroid-stimulating hormone, somatotrophs that secrete growth hormone, and lactotrophs that secrete prolactin (PRL) (18). The pituitary gland also contains pituicytes and folliculostellate cells (FSCs), as well as vascular pericytes and endothelial cells (19). Pituitary HPCs secrete their hormones by constitutive and regulated exocytosis, in response to several hypothalamic neurohormones (18) and endogenous ligands that act in an autocrine and/or paracrine fashion (20). The action of these ligands is mediated by G protein-coupled receptors (GPCRs) and receptor tyrosine kinases (RTKs), the activation of which leads to calcium mobilization from the ER. These cells are excitable and fire action potentials spontaneously or in response to activation of specific GPCRs, and the firing pattern and the accompanied pattern of calcium signaling are cell-type-specific (21).

## Synthesis and distribution of PIPs within the cell

Four PIKs contribute to the synthesis of PI4P: PI4KA, PI4KB, PI4K2A, and PI4K2B (**Figure 1B**). The conversion of PI4P to PI(4,5)P2 is mediated by three PI4P5Ks: PIP5K1A, PIP5K1B, and PIP5K1C, the latest having several splice forms. PI5P also contributes to the formation of PI(4,5)P2 by PIP4K2A/B, but probably generates only a small local pool of this messenger. PI(4,5)P2 is a substrate for class I PI3Ks, consisting of the catalytic units PI3KCA, PI3KCB, PI3KCG, and PI3KCD, each controlled by one of several regulatory subunits. PI3-phosphatases antagonize the action of PI3K by removing the 3-phosphate from PI(3,4,5)P3; PI5-phosphatases can remove phosphate from the 5-position of PI(3,4,5)P3, PI(4,5)P2, and PI(3,5)P2; and PI4-phosphatases remove phosphate from the 4-position of PI(3,4)P2 or PI(4,5)P2 (22). **Figure 1B** also summarizes the most used inhibitors of these PIKs.

Movements of PIPs are spatially restricted to PM and organelle membranes, where they are produced by enzymes associated with these membranes. PI4KA is predominantly localized in PM (23, 24), while PI4KB is localized on the Golgi apparatus (25, 26). PI4K2A is also present in the Golgi complex, as well as in the endosomal membrane and synaptic vesicles, and PI4K2B is localized to endosomal and perinuclear membranes (27–29); both type II PI4K enzymes have also been detected at PM (30). Three types of PIP5K1s are localized to PM controlling the conversion of PI4P to PI(4,5)P2 (31, 32). Class I PI3Ks are recruited to PM *via* RTKs or GPCRs, leading to the generation of PI(3,4,5)P3 and activation of Akt (33, 34). Therefore, PM is highly enriched in PI4P, PI(4,5)P2, and PI(3,4,5)P3 (12); PI4P is also present at high levels in the Golgi apparatus and the *trans*-Golgi network (35), and PI4P and PI(4,5)P2 in exocytic vesicles (9).

## PI(4,5)P2 and phospholipase C signaling

PI lipids were discovered nearly seventy years ago as a minor phospholipid species, whose turnover was activated by stimulation of hormone secretion (36). This was followed by the discovery of receptor-mediated activation of PLC, which hydrolyses PI(4,5)P2 into two intracellular messengers, inositol 1,4,5-trisphosphate (IP3) and diacylglycerol (**Figure 1C**). IP3 binds to its receptor located in the ER membrane and, together with calcium, controls its gating. Once activated, IP3 receptors function as calcium channels, allowing this cation to be released from the ER into the cytosol. Calcium flux through IP3 receptor channels can be terminated by their inactivation in a calcium-dependent manner. Thus, the IP3 branch of this signaling pathway leads to calcium mobilization, while the other branch follows the production of diacylglycerol, which together with calcium activates protein kinase C enzymes (17). Two families of calcium-mobilizing receptors, GPCRs and RTKs, drive the activation of different forms of PLC (37).

Pituitary cells express several genes encoding calcium-mobilizing GPCRs in a cell-type-specific manner: muscarinic receptors *Chrm1* and *Chrm3* (gonadotrophs, FSCs), angiotensin receptors *Agtr1a* (corticotrophs, FSCs) and *Agtr1b* (lactotrophs), purinergic receptors *P2ry1* (lactotrophs) and *P2ry2* (FSCs), endothelin receptors *Ednra* (gonadotrophs, lactotrophs, somatotrophs, FSCs) and *Ednrb* (FSCs, pituicytes), growth hormone secretagogue receptor *Ghsr* (somatotrophs), gonadotropin-releasing hormone receptor *Gnrhr* (gonadotrophs), serotonin receptor *Htr3a* (corticotrophs, melanotrophs, somatotrophs, pituicytes), thyrotropin-releasing hormone receptor *Trhr* (thyrotrophs, lactotrophs), vasopressin receptors *Avpr1a* (FSCs) and *Avpr1b* (corticotrophs) and PACAP receptors *Adcyap1r1* (FSCs, gonadotrophs) (18, 19). Pituitary cells are also equipped to signal *via* PLC gamma. The gene for this enzyme (*Plcg1*) is well expressed in all pituitary cells, as well as RTKs genes: *Fgfr1* (all cell types), *Fgfr2* and *Fgfr3* (FSCs, pituicytes); *Egfr* (all cell types), and *Erbb4* (FSCs and pituicytes) (19, 38).

Activation of these receptors in pituitary cells leads to a large and sudden increase in cytosolic calcium, followed by a non-oscillatory decay in calcium concentration forming a plateau phase (biphasic response), or an oscillatory change, with a frequency of 10-30 spikes per minute. For example, thyrotrophs and lactotrophs respond with biphasic calcium signals and hormone secretion to TRH administration (39, 40). In gonadotrophs, the initial rise in cytosolic calcium is usually followed by large calcium oscillations. The frequency of calcium spiking and the rate of secretion depend on the GnRH level, that is, there is frequency coding of calcium signaling and secretion. The oscillatory calcium response lasts for several hours during continuous GnRH receptor activation (41). In contrast, immortalized gonadotrophs respond to GnRH administration with non-oscillatory calcium signals, which desensitizes during sustain GnRH stimulation, reflecting a decrease in IP3 generation (42). Calcium is the main but not the only factor controlling the exocytosis of secretory vesicles; several other intracellular signaling pathways triggered by activation of calcium-mobilizing GPCRs contribute to the effectiveness of calcium-secretion coupling (18).

## PI(4,5)P2 and ion channel gating

It is well established that the PM level of PI(4,5)P2 is one of the key factors that modulates the function of voltage- and ligand-gated channels by interacting with the cytoplasmic domains of these proteins (43). Pituitary HPCs express a number of voltage- and ligand-gated ion channels and fire action potentials spontaneously and in response to application of Gq/11 and Gs-coupled receptor (18, 44) and activation of ligand-gated channels (45). These channels play an important role in pituitary functions, including hormone synthesis and secretion, but the role of PI(4,5)P2 in their gating has not been evaluated. Here, we list pituitary channels that have been shown to be regulated by PI(4,5)P2 in other cell types.

Depletion of PI(4,5)P2 in the PM results in the closure of voltage-gated potassium channels KCNQ1-4 and increased excitability of cells expressing these channels (46–48); pituitary HPCs express only *Kcnq2* gene (19). Inwardly-rectifying potassium channels also control the resting membrane potential and are regulated by PI(4,5)P2 (49, 50), and pituitary cells express *Kcnj3*, *Kcnj5*, *Kcnj6*, *Kcnj9*, and *Kcnj11* genes (19). The gating of big and small calcium-activated potassium channels also depends on PI(4,5)P2, keeping cells quiescent and hyperpolarized (51, 52). The *Kcnma1* gene encoding big conductance channels is expressed in all HPCs, and small apamin-sensitive conductance channels are expressed only in gonadotrophs and lactotrophs (18). Depletion of PI(4,5)P2 also reduced the current amplitudes of Cav1 and Cav2 calcium-conducting channels, with opposite physiological consequences: reduced calcium influx and calcium-dependent synaptic neurotransmitter release. Elevation in PI4P production did not restore the Cav channel conductance (53–55). These channels are well expressed in pituitary cells: *Cacna1a*, *Cacna1c*, *Cacna1d*, and *Cacna1h* in all HPCs, and *Cacna1g* only in lactotrophs and somatotrophs (19, 56). Pituitary cells also express ATP-gated P2X2, and P2X4 channels (45), which are known to be regulated by PI(4,5)P2 (57, 58).

The physiological mechanism of PI(4,5)P2 reduction in PM was also studied. It has been observed that the activation of PLC signaling pathways is accompanied with closure of PM ion channels affecting cell excitability. Sustained activation of Gq-coupled muscarinic receptors, closes partially open KCNQ channels (59). The same receptor-mediated PI(4,5)P2 depletion also reduced the current amplitudes of Cav1 and Cav2 calcium-conducting channels (60). The lack of experimental data on this topic in pituitary cells limits our understanding of the contribution of ion channel closure to receptor efficacy during continuous or repeated agonist applications, a question of great physiological and clinical importance.

## PI(4,5)P2 and the exocytic pathway

Hormone secretion by pituitary cells and other neuroendocrine cells is a multistep process, beginning with hormone synthesis in the ER, followed by transfer to the Golgi complexes for modification, sorting, and packaging into secretory vesicles, which bud from the trans face of the Golgi apparatus. Secretory vesicles then move along microtubules approaching PM for docking. The attached vesicles undergo priming to prepare for calcium-dependent fusion with PM, which occurs as complete fusion or as a kiss-and-run process. The first evidence for a direct role of inositol phospholipids in this multistep process comes from work of the Martin (61, 62) and Holz groups (63, 64) using adrenal neuroendocrine cells. Further work identified PI(4,5)P2 as the major PIP required for the exocytic process (65–68). The role of PI(4,5)P2 in exocytosis was further established by the finding that stimulation of PI4P5-kinases facilitated secretion (68) and knockout of PIP5K1C caused a reduction in secretory vesicle priming (69). PI(4,5)P2-dependent facilitation of secretion in mouse pituitary melanotrophs has also been reported (70).

During the last 10 years, several PI(4,5)P2-sensitive proteins involved in the control of exocytosis have been identified. Calcium-dependent activator protein for secretion (CAPS) is a dense-core secretory vesicles-bound protein (71), which plays a role in priming of secretory vesicles (67, 72). Rapid, regulated dense-core vesicle exocytosis in rat pituitary melanotrophs also requires the CAPS protein (73), suggesting that a similar role is played by PI(4,5)P2 in pituitary cells. Munc13 proteins (74) are also effectors for PI(4,5)P2 and contribute to the priming of secretory vesicles (75, 76). Synaptotagmin-1, another secretory vesicle protein, acts as a calcium-sensor for regulated exocytosis, requires PI(4,5)P2 to enhance its calcium sensitivity (77, 78), and contributes to docking, priming and fusion (79). PM-associated syntaxin-1 (80, 81) plays a critical PI(4,5)P2- and/or (PI(3,4,5)P3-dependent role in regulated exocytosis as a partner for SNAP-25, another PM associated SNARE protein (82–84). Together, these proteins contribute to secretory vesicle fusion. Spontaneous and stimulated PRL release from rat lactotrophs is also associated with PM regions enriched in SNARE proteins (85). For more details on PI(4,5)P2-dependency of the exocytic pathway see review (13).

## PI(4,5)P2 and PI3K signaling

Class I PI3Ks selectively recognize and phosphorylates PI(4,5)P2 to make PI(3,4,5)P3 (86). These enzymes are heterodimers of the p110 catalytic subunit closely associated with a regulatory subunit, which keeps the heterodimer catalytically inactive. There are four catalytic subunits and five regulatory subunits in class I PI3Ks (87). In rat pituitary cells, only the *Pik3ca* gene reaches the threshold for detection by scRNAseq, as do three regulatory subunit genes: *Pik3r1*, *Pik3r2*, and *Pik3r3* (14). In general, the RTK-mediated increases in PI3K activity led to the activation of the protein kinase, Akt, which in turn initiates a cascade of cellular responses. There are three isoforms of this enzyme: Akt1, Akt2, Akt3, which contribute to their diverse cellular roles (88). PI3K is “antagonized” by PTEN, which converts PI(3,4,5)P3 back to PI(4,5)P2 (89). The *Akt1*, *Akt2*, *Akt3*, and *Pten* genes are expressed in all pituitary cell types (19), suggesting the common operation of this signaling pathway in the gland.

The PI3K/Akt pathway is a key contributor to carcinogenesis in endocrine tissues, including pituitary cells (90). PI3K/Akt has also been implicated in the control of PRL release in mammalian lactotrophs (91). A dual regulatory effect of this pathway was reported: inhibition of basal PRL release and enhancement of PRL release in IGF-I-stimulated cells. A stimulatory role for PI3K in basal and GnRH-stimulated GH and LH release has also been shown in fish pituitary cells (92, 93). The status of electrical activity and calcium signaling was not assessed in these studies. However, experiments with αT3-1 and LβT2 immortalized mouse gonadotrophs revealed that wortmannin at a concentration that inhibits both PI3K and PI4K has no significant effect on GnRH-induced calcium mobilization, and that PI3K can influence the expression of gonadotroph-specific genes (94). In contrast, we observed no effect of the PI3K inhibitor LY294002 on PRL secretion (14). The observations that (PI(3,4,5)P3 and not PI(4,5)P2 shows a regulatory role on *Drosophila’s* synaptic vesicle exocytosis (95) may also explain the difference between fish and mammalian pituitary cells. However, in the work with the adrenal neuroendocrine cells also reached opposite conclusions; there was no major role for PI(3,4,5)P3 in the control of exocytic pathway (65, 96) compared to the inhibition of regulated exocytosis observed in LY294002-treated cells (97). This could be related to the concentrations of LY294002 used in the experiments, since this compound also inhibits PI4K at higher concentrations (98, 99).

## PI(4,5)P2-independent functions of PI4P

For many years, PI4P was thought to contribute to the control of cellular function just as the precursor of PI(4,5)P2 and PI(3,4,5)P3. A good example is the role of PI4KA in controlling the PI4P pool at PM, which is required for continuous activation of PLC (23, 24). However, more recently, a large body of evidence has accumulated showing that PI4P is also a direct regulator of cellular functions (100, 101) (**Figure 1C**). At PM this includes the role of PI4P as a substrate for PI(4,5)P2 at PLC (102, 103). Similarly, PI4P may contribute to the regulation of PM ion channel gating (104–107). PI4P has ability to recruit cytosolic signaling molecules containing PI4P-binding motifs. The role of PI4P in the Golgi compartment is well established (100). For example, depletion of PI4P by recruiting Sac1 to the Golgi inhibits cargo trafficking from the *trans*-Golgi network to PM and endosomes (108). Several studies have elucidated the role of PI4P in cargo budding and sorting, vesicle formation, fission and fusion, Golgi trafficking, and non-vesicular and lipid transport [reviewed in (101)]. PI4P may also play a role in trafficking at endosomes and lysosomes (109–111). Additionally, PI4P is required for the fusion of coat protein complex II vesicles from ER to Golgi compartments presumably by direct interaction with SNARE-dependent fusion (112). The physiological roles of PI4P were further supported using the cell-specific inactivation of *Pi4ka* and *Pi4kb* in conditional knockout mice (113, 114).

**Figure 2:**
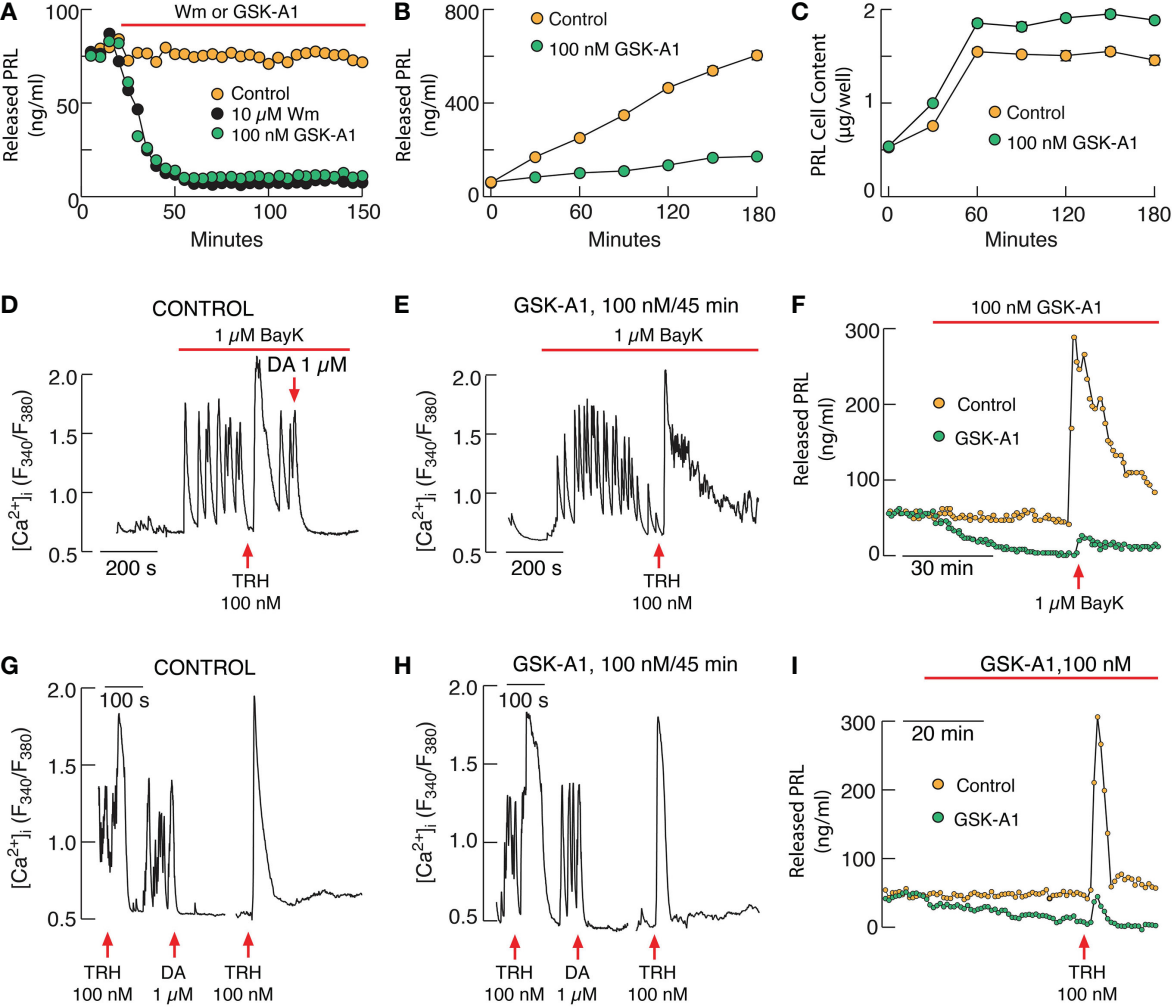
PI4KA controls basal and receptor-stimulated exocytosis in pituitary lactotrophs independently of PI(4,5)P2. **(A–C)**. Inhibition of basal prolactin (PRL) release by Wm and GSK-A1 in perfused **(A)** and static pituitary cells **(B)** without affecting *de novo* PRL synthesis **(C)**. **(D–F)** GSK-A1 also does not affect stimulated voltage-gated calcium influx by L-type calcium channel agonist BayK 8644 (BayK) in pituitary lactotrophs **(D, E)** but inhibits BayK-stimulated PRL release in perfused pituitary cells **(F)**. **(G–I)** GSK-A1 does not inhibit thyrotropin-releasing hormone (TRH)-stimulated calcium mobilization in pituitary lactotrophs **(G, H)** but inhibits basal and TRH-stimulated PRL release in perfused pituitary cells **(I)**. DA, dopamine. Derived from reference (14).

We recently assessed the role of PI4P in PRL secretion using cultured rat pituitary cells. Application of wortmannin at a concentration that inhibits both PI4KA and PI4KB and GSK-A1, a PI4KA inhibitor, completely blocked basal PRL secretion in perfused pituitary cells within 40-60 min application (**Figure 2A**). Inhibition was also observed in static cultures of pituitary cells (**Figure 2B**), without affecting *de novo* PRL synthesis (**Figure 2C**). In contrast, PIK93, an inhibitor of PI4KB, was ineffective (14). Basal PRL release is driven by spontaneous electrical activity and accompanied voltage-gated calcium influx (115), which was not affected by wortmannin and GSK-A1 treatments during 2-3 h administration (14). BayK 8644, an L-type calcium channel agonist, stimulated calcium influx was also not affected by GSK-A1 (**Figures 2D, E**), whereas BayK 8644-induced PRL release was blocked (**Figure 2F**). Similarly, TRH-stimulated calcium mobilization was not blocked (**Figures 2G, H**) in contrast to TRH-induced PRL release (**Figure 2I**). PIK93 did not mimic the effects of wortmannin and GSK-A1, further supporting a role of PI4KA in PRL secretion (14). These results indicate that inhibition of PRL release by depletion of PI4P at PM occurs downstream of calcium signaling. Because of the sensitivity of the SNARE complex to PI4P in fusion of intracellular vesicles between different intracellular organelles (112), it is reasonable to assume that the SNARE complex that mediates the fusion of secretory vesicles at PM is also sensitive to PI4P.

## Concluding remarks and future perspectives

Here, we briefly reviewed the literature showing that the multistep process of hormone secretion by neuroendocrine cells requires the presence PI(4,5)P2 either as a substrate for PLC- and PI3K-dependent pathways or playing a direct role in the exocytic process. These include the role of PI(4,5)P2 in calcium signaling, a critical step in regulated exocytosis, either as IP3-dependent calcium mobilization and/or voltage-dependent calcium influx, priming of secretory vesicles by CAPS and Munc13, and assembly of SNARE proteins sintaxin-1 and SNAP25, for secretory vesicle fusion. Data also suggests PI(4,5)P2-specific and indiscriminate roles of PIPs in hormone secretion; PI4P could substitute for PI(4,5)P2 in PLC activation and gating of some channels (**Figure 1D**), and both PI(4,5)P2 and PI(3,4,5)P3 have been implicated in syntaxin-1 clustering. Our recent study suggests a possible role for PI4P downstream of calcium signaling, raising the possibility that it also plas a key role in SNARE function. Additional studies are needed to identify the primary and secondary roles of specific PIPs in the control of a particular step of exocytosis. These include elucidating the kinetics of PIP pool depletion under physiological and pharmacological conditions. The use of kinases inhibitors, especially wortmannin and LY294002, requires attention to distinguish between PI4Ks and class I PI3K signaling pathways.

In general, the roles of PI(4,5)P2 in PLC and PI3K actions in HPCs are sufficiently well characterized. However, that is not the case for direct effects of PIPs on signaling and exocytic pathways. These include the need to characterize the PIP-dependent properties of pituitary voltage- and ligand-gated channels and their calcium signaling functions using well-established protocols for these studies. Also, pituitary cells provide suitable experimental models to address specific questions related to the role of PIPs in hormone secretions. For example, the contribution of PIPs to the desensitization of stimulus-secretion coupling during sustained activation of Gq/11-coupled GPCRs and the relevance of the calcium signaling pattern in this process. Certainly, cell type-specific knockout of the genes encoding these kinases could provide additional insight into the role of specific PIPs in cellular functions, including hormone secretion. Considering the novel finding of the possible role of PI4P in PRL secretion, this line of future research should include lactotroph-specific knockouts of *Pi4ka* and *Pi4kb*.

## Author contributions

All authors contributed to the article and approved the submitted version.
